# Idiopathic unilateral third nerve palsy with pupillary sparring in 10-year-old child -a case report

**DOI:** 10.1016/j.amsu.2022.104723

**Published:** 2022-09-16

**Authors:** Qaisar Ali Khan, Sohail Adnan, Naseer Ahmad, Hassan Mumtaz, Ravina Verma, Alishba shahi, Ameena Shahi, Sumaira Iram, Abdul Baqi

**Affiliations:** a DHQ and Teaching Hospital KDA Kohat, Khyber, Pakhtunkhwa, Pakistan; b St. George's University Grenada, West indies, Grenada; c Clinical Research Associate, Maroof International Hospital, Public Health Scholar: Health Services Academy, Pakistan; d Lady Reading Hospital Peshawar, Khyber-Pakhtunkhwa, Pakistan; e Sultan Qaboos University and Hospital, Muscat, Oman; f Mercy Saint Vincent Medical Centre Toledo, Ohio, United States

**Keywords:** Idiopathic, Nerve palsy, Congenital, Ptosis

## Abstract

**Introduction and importance:**

Aneurysm, diabetes mellitus, central nervous system (CNS) infections, pituitary tumors, and ischemia alterations are all potential causes of unilateral oculomotor nerve palsy, a common clinical disease.

**Case presentation:**

A 10-year-old child presented with right eyelid ptosis and restricted eye movements associated with diplopia and pain in the right eye. Brain imaging and laboratory tests revealed no obstruction, infection, or hypercoagulable state. The condition was labeled as idiopathic. A patient was diagnosed with ptosis through a sling procedure and after 2 and 4 weeks of follow-up was told he had mild anemia. The patient was prescribed ferrous sulfate 8mg once daily for 4 months and his condition improved.

**Clinical discussion:**

Surgery can correct the appearance of crossed eyes, but it seldom restores or significantly improves binocular function. Amblyopia and the loss of binocular vision can occur in children with third nerve palsy due to the excessive angle of incitant strabismus and the resulting ptosis.

**Conclusion:**

Patients with idiopathic third nerve palsy must be informed of their prognosis so that they can make an informed decision about whether or not to undergo surgery. Clinical examination is the only way to identify a child's condition and proper investigations and a full history of prenatal and antenatal courses are required.

## Highlights

• Clinical examination is the only way to identify a child with idiopathic third nerve palsy.
• Proper investigations and a full history of prenatal and antenatal courses are required to exclude congenital and acquired reasons.
• Patients must be informed of their prognosis so that they can make an informed decision about whether or not to go through with the procedure.

## 1 Introduction

Oculomotor palsy has both congenital and acquired causes. Congenital causes include intrauterine infections and trauma during delivery [1]. Among acquired causes trauma is the most common cause, other causes include intracranial neoplasia, meningitis, encephalitis cerebral artery aneurysm, and blockage of the small vasculature which supplies the nerve fibers due to a hypercoagulable state.

We present a case of unilateral third nerve palsy which developed at 4 months of age. Initially, the patient had mild ptosis, but it progresses over the past few years, patient's birth and past medical history were unrevealing. Brain imaging and laboratory tests didn't reveal the exact cause and the condition was labeled as idiopathic. Surgery for ptosis was performed and the patient was instructed to follow up.

## 2 Case report

A 10-year-old boy presented to the outpatient department with right eye ptosis, restricted eye movements, and diplopia. It developed at 4 months of age and was acute in onset, but the mother notices it a few weeks later. Initially, it was mild but right eye ptosis progressively worsened over the past few years until the patient has difficulty opening the right eye. The patient had not progressively fatigability over the course of the day.

**Fig. 1:**
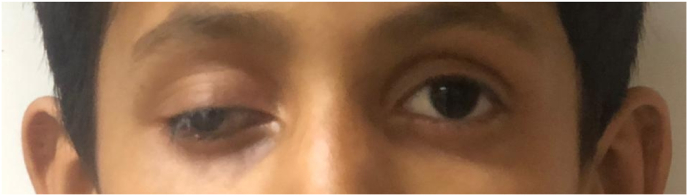
**Shows ptosis of the right eye**.

The consultant ophthalmologist noted no aggravating or relieving factors. Ptosis was associated with pain and tearing in the right eye periodically, as shown in Fig. 1. The patient was born full-term via normal vaginal delivery in the hospital. The patient prenatal and antenatal course was uncomplicated, and the mother took regular prenatal care. The patient is fully vaccinated, and his past medical and surgical history was not significant. The systemic review was unremarkable for any central nervous system (CNS), cardiovascular or systemic illness. Family history was significant for bilateral cataracts in paternal grandfather at the age of 65.

No Drug History or Allergies was reported was by the family.

Ophthalmological examination was done and findings have been shown in Table 1.

**Table 1:** Ophthalmological examination findings.

	Right eye	Left eye
Upper eyelid	Drooping until covering the limbus	Normal
Sclera	Normal	Normal
Cornea	Normal	Normal
Pupil size	3mm	4mm
Direct light reflex	Sluggish	Normal
Consensual light reflex	Sluggish	Normal
Accommodation reflex	Impaired	Normal
Fundus	Normal	Normal
Visual acuity	6/6	6/6
Medial movement	Restricted	Intact
Lateral movement	Intact	Intact
Downward movement	Restricted	Intact
Upward movement	Intact	Intact
Repetitive nerve stimulation test	Negative	Negative

mm; millimeter.

Initial laboratory investigation including complete blood count, C reactive protein, and blood random sugar was unremarkable, as shown in Table 2.

**Table 2:** Initial laboratory investigations.

Investigation	Result	Unit	Reference range
Hemoglobin	11.6	g/dl	13–17.5
White blood cell count	4650	Cells/mcL	4500–11000
Red blood cell count	3,45,000	Cells/mcL	450000–600000
Platelet count	2,92,000	Cells/mcL	1,50,000–4,50,000
Mean corpuscular volume	77	Fl	80–100
Mean corpuscular hemoglobin concentration	34.2	g/dl	32–36
RDW	48.4	%	12–16
CRP	24		
Blood random sugar	86	mg/dl	80–126

ESR = Erythrocyte sedimentation rate, RDW = red cell distribution width.

**Fig. 2:**
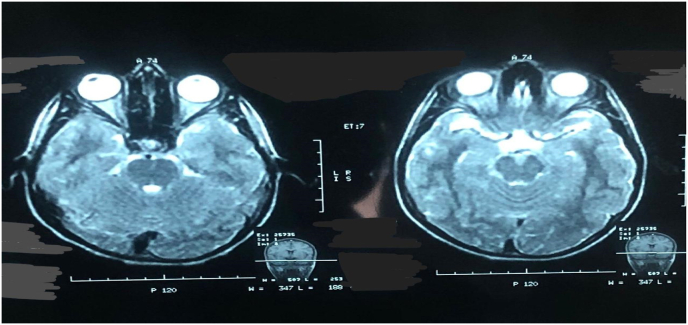
Magnetic Resonance imaging shows a normal study with no obstructive lesion in the orbit and anterior cranial fossa.

Magnetic resonance imaging (MRI) brain has been ordered for intracranial anomalies reporting no obstructive lesion in the orbit and anterior cranial fossa as given in Fig. 2.

Further tests were ordered for hypercoagulability and systemic illness and the results have been shown in Table 3.

**Table 3:** Hypercoagulability & Systemic illness Results.

Investigation	Result	unit	Reference range
ANA	1:40 (negative)		
Protein C	324.1	%	72–160
Protein S	81.7	%	60–150
Antithrombin 3	96	%	80–120
Factor V laden mutation	Negative		

A high protein c level is not significant in the case of children. Despite the detailed history and laboratory workup, the exact cause of the disease remained unknown, and the condition has been labeled as idiopathic. Patching of the right eye has been done but the condition did not improve, patient family was interested in surgical procedures for the condition. The patient was operated on for ptosis through a sling procedure and the patient was called for follow-up after 2 and 4 weeks. As a low level of hemoglobin suggested mild anemia the patient was prescribed ferrous sulfate 8mg once daily for 4 months.

At 4 weeks follow up patient diplopia was relieved up to some extent and the patient was instructed for regular follow-up.

Our Case Report is in compliant with SCARE 2020 Guidelines [2]. A complete SCARE 2020 checklist has been provided as a supplementary file.

## 3 Discussion

Cranial nerve III palsies, also known as oculomotor nerve palsy, may result from various causes; however, the etiology remains unknown in some instances. Nerve palsies less frequently occur in children with an incidence rate of 7.6 per 10, 00 000 [3]. Idiopathic causes of unilateral nerve palsy remain unsolved, accounting for 12–14% of cases in children [4,5] despite recent breakthroughs in imaging and laboratory procedures. Congenital and acquired causes are involved. Unilateral and total congenital oculomotor nerve palsy are the most common outcomes. Symptoms of a paralyzed ocular reflex might vary widely. Often, the palsy goes unnoticed at the time of birth. When it comes to eye-movement disorders, the majority of them are caused by an unknown source, but a few of them are inherited or caused by orbital damage [6].

The patient's history and physical examination revealed no evidence of an underlying cause in our case study. Except for the medial movement of the right eye, which indicated pupil sparing palsy, the patient exhibited normal visual acuity and color vision in both eyes. Pupil sparing oculomotor palsy due to an unknown etiology is extremely uncommon in children.

Oculomotor nerve palsies (OMNPs) that spare the pupil but are otherwise complete are typically caused by ischemia, whereas OMNPs that spare the pupil but are otherwise incomplete require evaluation for a compressive or infiltrative process. It's worth noting that Lust Bader and Miller have reported an instance of a full, pupil-preserving OMNP produced by a basilar tip aneurysm. Obstructive lesions and prenatal infection can cause bilateral cranial nerve palsies, which have been linked to other cranial nerve palsies, such as the fourth and sixth. A compressive lesion isn't necessarily caused by frequent pupil involvement in youngsters, as it is in adults [7].

The cause of five children's third nerve palsy was never determined in a study done in 1987 by Keith [8]. However, studies on pediatric third nerve palsy are few and far between, but those that have been done suggest that idiopathic cases of this nerve palsy are often linked to myasthenia gravis or multiple sclerosis and require more time to follow up on. However, it may be accompanied by abnormal regeneration, cranial nerve palsies, central nervous system abnormalities or developmental delays. Eyelid drooping, ophthalmoplegia, and dilation of the pupil are all symptoms of third nerve palsy. When you try to abduct your head or look downward, your lids may retract, which could be a sign of abnormal regeneration [9,10].

The management of patients with oculomotor nerve palsy is one of the most challenging issues for the strabismus surgeon. Each patient has a different presentation depending on the extent of the paresis, recovery, and presence of aberrant regeneration or other associated factors. Therefore, the management of every patient varies accordingly. In children, the presence of amblyopia and loss of binocularity, due to the large angle of incitant strabismus and associated ptosis further complicate the management of third nerve palsy. Surgery can cosmetically align the eyes, but it rarely returns or achieves significant binocular function [11].

## 4 Conclusion

Clinical examination is the only way to identify a child with idiopathic third nerve palsy. Before designating it idiopathic, proper investigations and a full history of prenatal and antenatal courses are required to exclude congenital and acquired reasons. Before undergoing surgery, patients must be informed of their prognosis so that they can make an informed decision about whether or not to go through with the procedure.

## Ethical approval

Not needed for case report.

## Please state any sources of funding for your research

Nill.

## Author contribution

Contributorship Statement:

1. The main concept was determined by Qaisar Ali Khan, Naseer Ahmad
2. Collection of data is done by Sohail Adnan, Ameena Shahi
3. Writing of the manuscript is done by Ravina Verma, Alishba shahi, Sumaira Iram
4. Manuscript editing is done by Hassan Mumtaz, Abdul Baqi

## Registration of research studies

1. Name of the registry: Research Registry
2. Unique Identifying number or registration ID: researchregistry8293
3. Browse the Registry - Research Registry

## Guarantor

Qaisar Ali Khan.

## Consent

The informed consent from the patient's guardian was obtained considering Helsinki's Declaration.

## Provenance and peer review

Not commissioned, externally peer reviewed.

## Patient perspective

If I hadn't delayed proper treatment, I would have enjoyed my life pain free.

## Declaration of competing interest

Nill.
